# 2,4,6-Trinitrotoluene Induces Apoptosis via ROS-Regulated Mitochondrial Dysfunction and Endoplasmic Reticulum Stress in HepG2 and Hep3B Cells

**DOI:** 10.1038/s41598-017-08308-z

**Published:** 2017-08-15

**Authors:** Hung-Yu Liao, Chih-Ming Kao, Chao-Ling Yao, Po-Wei Chiu, Chun-Chen Yao, Ssu-Ching Chen

**Affiliations:** 1 Department of Life Sciences, National Central University, no. 300, Jhing-da Rd., Jhongli City, Taoyuan, 32001 Taiwan; 2 Institute of Environmental Engineering, National Sun Yat-Sen University, no. 70, Lien-hai Rd., Kaohsiung, 80424 Taiwan; 3 Department of Chemical Engineering and Materials Science, Yuan Ze University, no. 135, Yuan-tung Rd., Jhongli City, Taoyuan, 32003 Taiwan; 4 College of Medicine, National Cheng Kung University, no. 1, University Road, Tainan, 701 Taiwan; 5 College of Medicine, National Taiwan University, no. 1, Jen-Ai Road, First Section, Taipei, 100 Taiwan

## Abstract

2,4,6-trinitrotoluene (TNT) has been reported to cause numerous adverse effects. However, the detailed molecular mechanisms underlying TNT-induced liver toxicity need to be elucidated. In this study, we used HepG2 (p53wt) and Hep3B (p53null) cell lines to investigate the cytotoxic effects of TNT. At first, we found that TNT significantly decreased cell viability and induced DNA damage. Thereafter, through transcriptomic analysis, we observed that the diverse biological functions affected included mitochondrial dysfunction and endoplasmic reticulum (ER) stress. Mitochondrial dysfunction was evidenced by the loss of mitochondrial membrane potential, increased expression of cleaved-caspase-9&-3 and increased caspase-3/7 activity, indicating that apoptosis had occurred. In addition, the expressions of some ER stress-related proteins had increased. Next, we investigated the role of reactive oxygen species (ROS) in TNT-induced cellular toxicity. The levels of DNA damage, mitochondrial dysfunction, ER stress and apoptosis were alleviated when the cells were pretreated with N-acetyl-cysteine (NAC). These results indicated that TNT caused the ROS dependent apoptosis via ER stress and mitochondrial dysfunction. Finally, the cells transfected with CHOP siRNA significantly reversed the TNT-induced apoptosis, which indicated that ER stress led to apoptosis. Overall, we examined TNT-induced apoptosis via ROS dependent mitochondrial dysfunction and ER stress in HepG2 and Hep3B cells.

## Introduction

2,4,6-trinitrotoluene (TNT) has been commonly used as an explosive throughout the world, and it is one of the most serious environmental contaminants in military sites where munitions were manufactured^1^. TNT has been shown to be highly toxic, mutagenic, and carcinogenic in some bacterial and animal tests^2–5^. In addition, TNT could lead to numerous adverse effects, including upper respiratory problems, gastrointestinal complaints, anemia, liver function abnormalities, and aplastic anemia^6, 7^. In China, a survey study of male workers from 8 Chinese military factories who were exposed to TNT for more than a year confirmed that TNT could increase the relative risk of 80%, especially liver cancer^8^. More recently, multiple studies have indicated that TNT-induced stress, including endoplasmic reticulum (ER) stress and oxidative stress, may lead to liver injury^7, 9^. However, the molecular mechanisms involved in stress-induced hepatotoxicity are still unclear, although some studies have shown that ER stress and the apoptotic pathway are involved in TNT-induced hepatic toxicity^7, 9, 10^. Noticeably, the role of reactive oxygen species (ROS) in mediating ER and mitochondrial stress needs to be fully investigated.

ROS profoundly impact a number of cellular responses such as DNA damage, cell cycle progression, and apoptotic cell death^11–13^. In eukaryotic cells, the mitochondrial electron transport is the main source of ROS during normal metabolism^12^. Excessive or sustained ROS can cause damage to proteins and DNA via diverse mechanisms, thereby activating or inhibiting the related signaling pathway^14^.

The ER plays an important role in chemical toxicant-induced apoptosis^15^. The ER is an organelle that maintains intracellular calcium homeostasis, protein synthesis, post-translational modification and proper protein folding^16^. A disturbance of ER Ca^2+^ homeostasis or the protein process can lead to ER stress, which in turn induces the production of ROS in the ER and mitochondria^17^. High ROS generation within mitochondria induces the opening of the mitochondrial permeability transition pore (mPTP)^17^. Subsequently, a number of proteins that regulate apoptosis become involved, contributing to cell death.

To determine the possibility of ROS involvement in apoptosis as described above, we detected ROS generation in cells by activating the mitochondrial and ER stress pathways. Further investigations into the links between ROS increase, DNA damage and apoptosis induced by ROS were also conducted. In this study, we investigated the detailed mechanisms underlying TNT toxicity in HepG2 cells. Furthermore, we investigated the effects of TNT toxicity in Hep3B cells and aimed to understand if the mechanisms of TNT toxicity in different human hepatoma cells were different based on the presence of p53 in HepG2 cells but not in Hep3B cells.

## Results

### Effects of TNT on cell viability, DNA damage and the activation of caspase-3/7 in HepG2 and Hep3B cells

**Figure 1:**
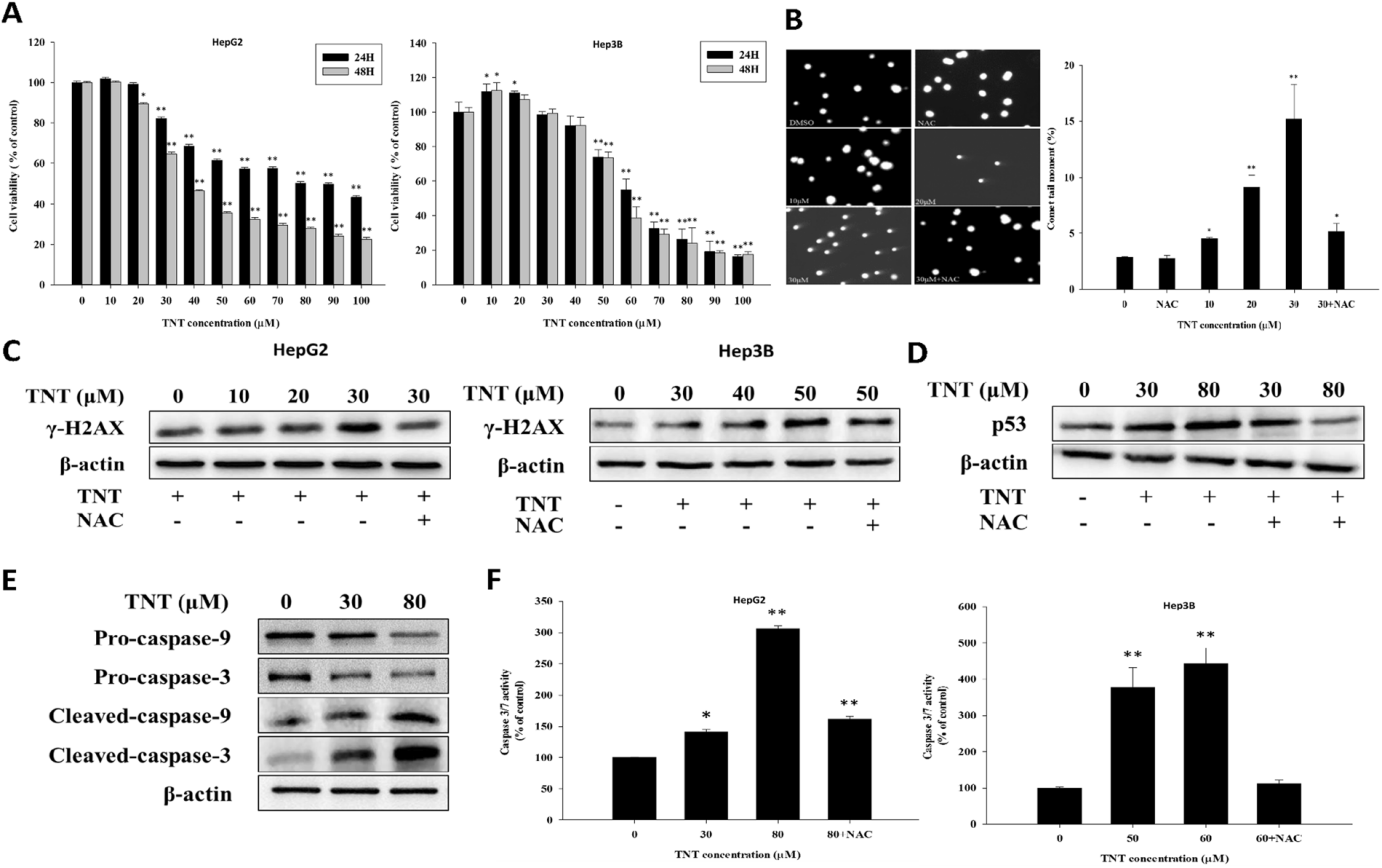
TNT-induced cytotoxicity, DNA damage and apoptosis in HepG2 cells in a dose- and-time dependent manner. (**A**) HepG2 and Hep3B cells were treated with TNT (0–100 μM) for 24 h and 48 h, and cell viability was analyzed by CCK-8 assay. (**B**) DNA damage was determined by comet assay in HepG2 cells treated with TNT for 24 h. (**C**) Effects of TNT on γ-H2AX protein expressions in HepG2 and Hep3B cells. The cells were exposed to TNT for 24 h. The protein expression of γ-H2AX was analyzed by Western blot. (**D**) Effects of TNT on p53 protein expressions in HepG2 cells. The cells were exposed to TNT for 24 h. The protein expression of p53 was analyzed by Western blot. (**E**) Effects of TNT on protein expression of pro-caspase-9 and -3 and cleaved-caspase-9 and -3 in HepG2 cells. The cells were exposed to TNT (0, 30, and 80 μM) for 24 h. The protein expression was analyzed by Western blot. (**F**) The caspase-3/7 activity was assayed after the HepG2 and Hep3B cells were treated with TNT and with or without NAC for 24 h via microplate reader. The data are presented as mean ± SD for four independent experiments with triplicate determinations. *P < 0.05; **P < 0.01 as compared with control.

To investigate the extent of the effect of TNT on HepG2 and Hep3B cells, we performed dose response or time course analysis of TNT-mediated proliferation inhibition, DNA damage and the activation of caspase-3/7 in HepG2 and Hep3B cells. We performed a CCK-8 assay to detect the level of cytotoxicity in TNT treated cells. The results show that TNT exhibited the cytotoxicity against the growth of cells in terms of dose response and time. Cell viability was reduced to about 50% after the cells were treated with TNT (80 μM) for 24 h in HepG2, and treated with TNT (60 μM) for 24 h in Hep3B (Fig. 1A).

To evaluate the ability of TNT to trigger genotoxic damage in hepatocytes, the HepG2 cells were first treated with different concentrations of TNT (in the range of 0 to 30 μM) for 24 h, and they were subsequently analyzed using an alkaline comet assay (Fig. 1B); the presence of a DNA-forming tail-like structure in a concentration-dependent manner was observed in cells treated with TNT. Second, HepG2 and Hep3B cells were treated with different concentrations of TNT (HepG2: 0–30 μM; Hep3B: 0–50 μM) for 24 h to determine the induction of protein related to the DNA damage marker: γ-H2AX^18^. We found that TNT induces γ-H2AX in a concentration-dependent manner in HepG2 and Hep3B cells (Fig. 1C). Third, we investigated the effect of TNT on p53 protein levels. We found that TNT significantly increases the expression of p53 as compared to HepG2 cells without added TNT (Fig. 1D).

As caspase cascade activation is a key event in apoptosis pathways, we found that TNT (30 μM and 80 μM) treatment with HepG2 reduced the expression of pro-caspase-3 and -9 and increased cleaved caspase-3 and -9, respectively, indicating that the treated cells induced apoptosis (Fig. 1E). This assumption was further substantiated by caspase-3/7 activity, which is an indicator of cell death (Fig. 1F). Our results demonstrate that TNT exposure induces significant DNA damage and apoptosis in HepG2 and Hep3B cells.

### Transcriptome analysis of TNT-induced impaired biological function and/or pathways

In order to systematically understand the transcriptional response from HepG2 cells treated with 30 μM and 80 μM TNT for 24 h, we first used RNA-seq, a high throughput method, to detect the differential genes (> or <2-fold, *P* < 0.05) in cells before and after 30 μM and 80 μM TNT treatments. These differential genes detected in 30 μM or 80 μM TNT are listed in Supplementary Tables S1 and S2. To validate the RNA-seq results in this study, we selected several differential expressed genes (DEGs) of particular relevance to oxidative phosphorylation/protein processing in the endoplasmic reticulum during the 80 μM TNT treatment and sought to corroborate the RNA-seq data using quantitative RT-PCR (Supplementary Table S3). The results showed a strong correlation between the RNA-seq and qRT-PCR data, which strongly confirmed that the RNA-seq results in this study are reliable. In order to better understand the functions of differentially expressed genes, we subjected the data to analysis for relationships as revealed by the Kyoto Encyclopedia of Genes and Genomes (KEGG) pathway, Using KEGG analysis, differential genes were identified with biological pathways/functions that were related to oxidative phosphorylation, the metabolic pathway and protein processing in the endoplasmic reticulum. In addition, Alzheimer’s disease, Huntington’s disease, and Parkinson’s disease were detected irrespective of the 30 μM and 80 μM TNT treatments (Supplementary Tables S4 and S5).

### TNT-induced ER stress in HepG2 cells

**Figure 2:**
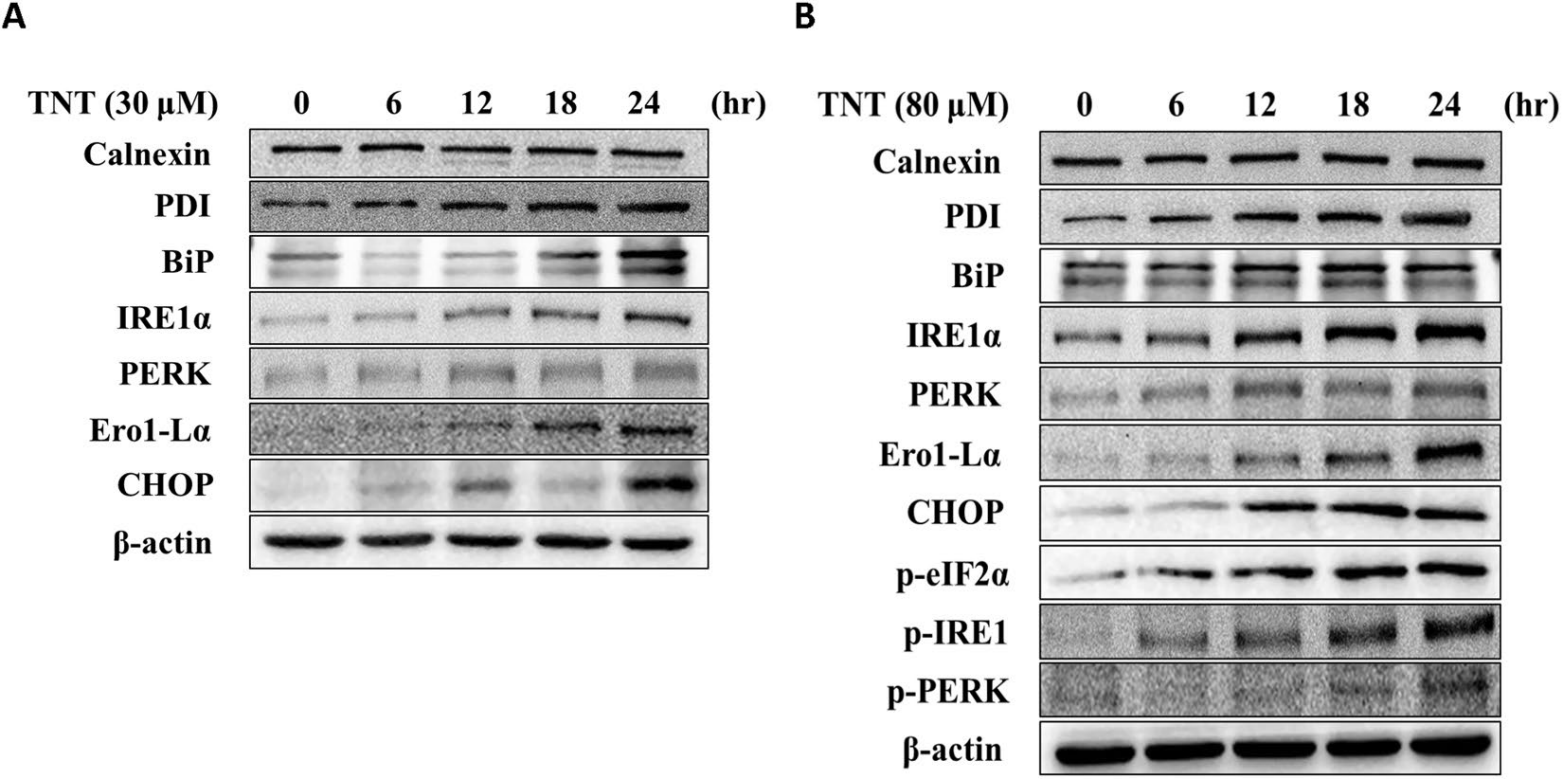
TNT-induced ER stress in HepG2 cells. The effects of (**A**) 30 μM TNT and (**B**) 80 μM TNT on the expression of ER stress related proteins. HepG2cells were treated with TNT at the indicated concentrations for 0, 6, 12, 18, and 24 h. Whole-cell lysates were obtained and subjected to Western blot analysis using antibodies against calnexin, PDI, BIP, IRE1-α, PERK, Ero1-Lα, CHOP, phosphorylated eIF2α, phosphorylated IRE1, phosphorylated PERK and β-actin. The bands were excised from different gels that were run under the same electrophoresis condition.

The altered redox homeostasis in the cell could cause ER stress, which in turn could induce the production of ROS in the ER and mitochondria^12^. To delineate the induction of ER stress by TNT in HepG2 cells, we investigated the induction of proteins related to ER stress after the 30 μM and 80 μM TNT treatments in the HepG2 cells. As shown in Fig. 2A and B, the expression of binding immunoglobulin protein (BiP) and protein disulfide isomerase (PDI) increased after the TNT treatment, but no differences were found in calnexin expression. The up-regulation of ER stress transducers inositol-requiring protein 1 alpha (IRE1-α) and PKR-like ER protein kinase (PERK) was observed. The dose response and time of C/EBP homologous protein (CHOP), transcriptional target (ER oxidase 1-like α [Erol-Lα]), phosphorylated eukaryotic initiation factor 2 (eIF2α), phosphorylated IRE1, and phosphorylated PERK also increased after the TNT treatment. We observed similar results in the Hep3B cells (Supplementary Fig. S1). These results indicated that TNT is capable of inducing ER stress in human hepatoma cells.

### TNT-induced apoptosis through mitochondrial dysfunction pathway

**Figure 3:**
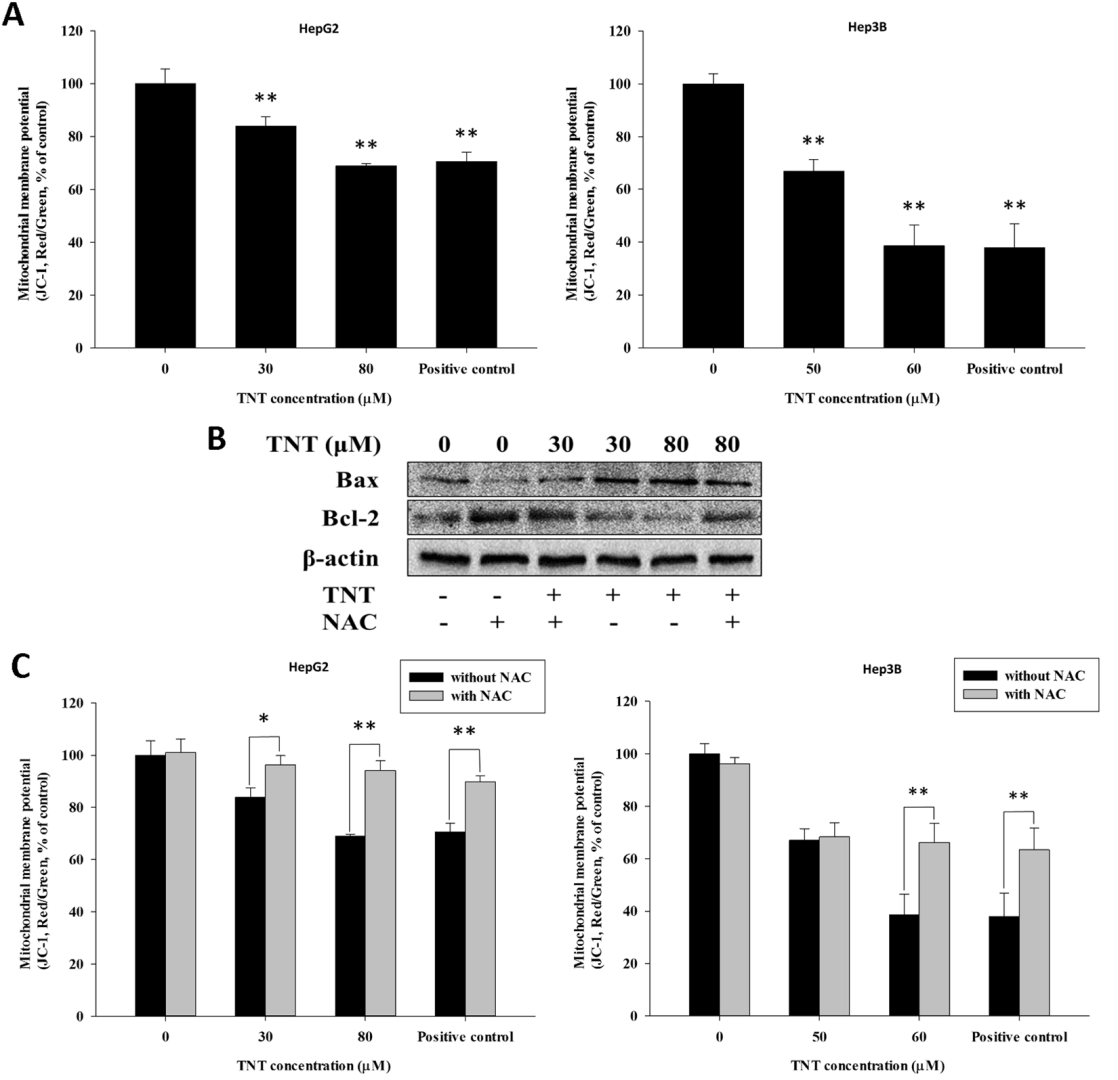
TNT triggered the mitochondrial dysfunction and apoptosis pathways. (**A**) TNT (0, 30, and 80 μM) caused a disruption of MMP in HepG2 cells, and TNT (0, 50, and 60 μM) caused a disruption of MMP in Hep3B cells after treatment for 24 h, as evidenced by an increased proportion of cells with green fluorescent light and a decrease proportion of cells with a higher red (JC-1 aggregates)/green (JC-1 monomers) ratio of JC-1 fluorescence. (**B**) TNT-induced mitochondrial dysfunction, as evidenced by Western blot analysis using antibodies against Bax, Bcl-2, and β-actin. (**C**) The cells were treated with different concentrations of TNT for 90 min in the presence or absence of 10 mM NAC, and the dissipation of MMP was measured. (the positive control: 100 μM H_2_O_2_)

ROS formation from mitochondria dysfunction would cause the mitochondrial membrane potential (MMP) to collapse completely^19, 20^. To investigate the effect of TNT on mitochondrial function, we first measured MMP using JC-1 dye. This fluorescent dye can spread selectively within the mitochondria depending on the membrane potential^12^. The exposure of HepG2 cells (30 μM or 80 μM) and Hep3B cells (50 μM or 60 μM) to TNT for 24 h caused disruption of the MMP, which was detected by a decrease in the red/green ratio of JC-1 fluorescence, as shown in the results of the cells treated with 100 μM H_2_O_2_ (the positive control; Fig. 3A). The loss of MMP was always accompanied by the mitochondrially mediated apoptotic pathway in human cells, as reported by Zhou *et al*.^12^. Additionally, MMP is a highly regulated process that is primarily controlled through interactions between pro- and anti-apoptotic members of the B-cell lymphoma 2 (Bcl-2) family^21^. Accordingly, we measured the expression of Bcl-2-associated X protein (Bax) and Bcl-2 using Western blot analysis (Fig. 3B). The results showed a dose-dependent suppression of Bcl-2 expression, but the expression of Bax exhibited the opposite results. These results suggest that the mitochondrially mediated apoptotic pathway was stimulated by TNT.

### The role of ROS formation in inducing DNA damage, ER stress, mitochondrial dysfunction, apoptosis, and cell death

**Figure 4:**
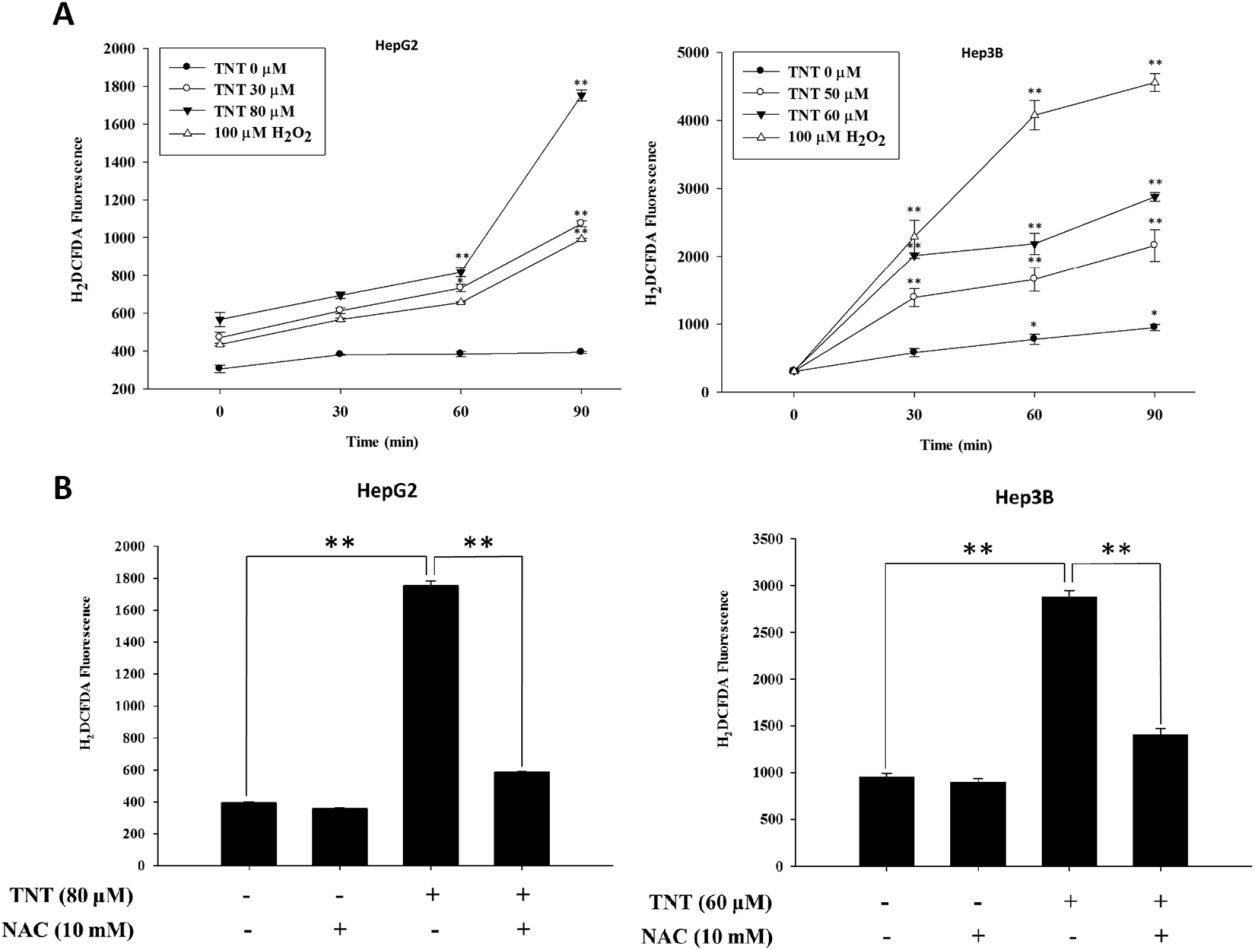
TNT induced the generation of ROS. (**A**) ROS expression levels of HepG2 and Hep3B cells exposed to different doses of TNT for 0 to 90 min. (**B**) The HepG2 cells were treated with 80 μM TNT, and the Hep3B cells were treated with 60 μM TNT, both for 90 min in the presence or absence of 10 mM NAC. The values represent the mean ± SD and were derived from at least three independent experiments. Triplicate measurements were performed for each experiment. *P < 0.05; **P < 0.01 (vs. control cells).

**Figure 5:**
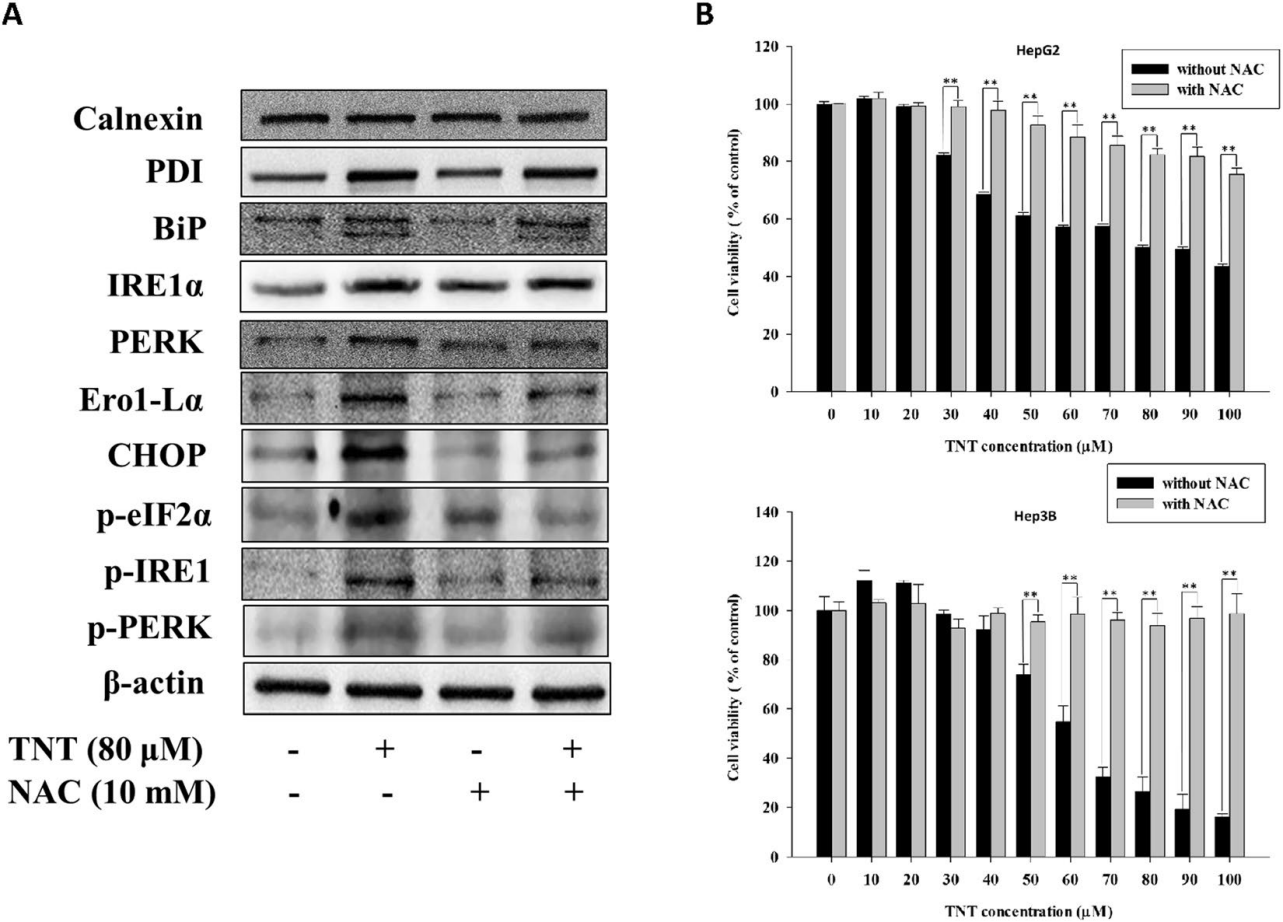
ROS-dependent ER stress was involved in the TNT-mediated ROS production. (**A**) HepG2 cells were treated with 80 μM TNT for 24 h in the presence or absence of 10 mM NAC, given the expression of ER stress-related proteins. The bands were excised from different gels that were run under the same electrophoresis condition. (**B**) The cells were treated with 0–100 μM TNT for 24 h in the presence or absence of 10 mM NAC, and then the cell viability was determined. The values represent the mean ± SD and were derived from at least three independent experiments. Triplicate measurements were performed for each experiment. **P < 0.01 (vs. control cells).

**Figure 6:**
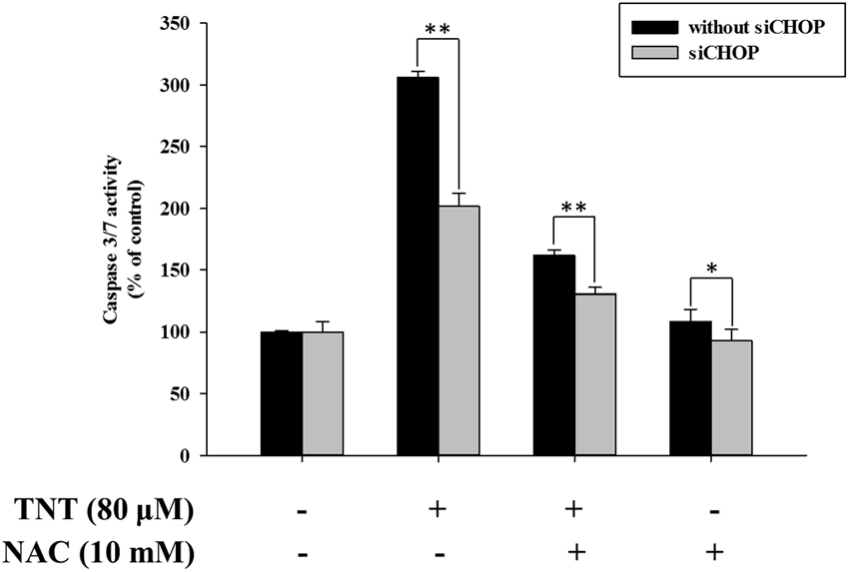
Effects of CHOP siRNA on TNT-induced ER stress in HepG2 cells. The cells were transfected with or without CHOP siRNA and treated with or without TNT (80 μM) and with or without NAC for 24 h. Caspase-3/7 activity was detected via microplate reader. The data are presented as mean ± SD for four independent experiments with triplicate determinations. *P < 0.05; **P < 0.01 as compared with control.

There has been much evidence to support the important role of ROS in initiating cascades of cell death^22^. We proved that TNT indeed generates ROS (Fig. 4A and B). To assess the effect of TNT on oxidative DNA damage, NAC—the antioxidant and ROS scavenger— was used for the functional role in TNT-induced DNA damage. The results revealed that the level of DNA damage was reduced in HepG2 and Hep3B cells after the addition of NAC, as compared to the control group (Fig. 1B–D). We indicated that TNT could result in oxidative DNA damage. We also tested the effect of NAC on ER stress, mitochondrial dysfunction, apoptosis, and cell viability. The pretreatment of cells with NAC decreased the ratio of expressed Bax to expressed Bcl-2 (Fig. 3B) Furthermore, NAC significantly blocked TNT-induced MMP loss (Fig. 3C), indicating that the process of mitochondrial-related apoptosis was inhibited (Fig. 1F). Lastly, we showed that NAC attenuated TNT-induced ER stress and cytotoxicity (Fig. 5A,B and Supplementary Fig. S1). These findings strongly suggest that TNT induces oxidative stress and generating oxidative relation lesions. Additionally, CHOP was the best characterized apoptosis factor related to the ER stress pathway. We decreased the expression of CHOP protein using the siRNA method (80% knockdown efficiency; Supplementary Fig. S2) to investigate whether ER stress induced apoptosis. The results revealed that apoptosis was decreased when the expression of CHOP protein was inhibited in HepG2 cells (Fig. 6). Overall, ROS formation elicited apoptosis through ER stress and mitochondrial dysfunction.

## Discussion

Cultures of HepG2 (p53wt) and Hep3B (p53null) cells are frequently used in *in vitro* models for human biotransformation in the human liver and is of great importance for toxicological and pharmaceutical studies^23^. Qiu *et al*. reported that p53 may not be the crucial factor to determine the differences in biological responses between HepG2 and Hep3B cell lines in confronting toxicants^24^. HepG2 cells of human origin retained active xenobiotic metabolizing enzymes and genotoxic sensitivity. Furthermore, HepG2 cells with the enzyme activity, and gene expression were similar to normal human liver cells^25, 26^. The use of HepG2 cells as the target for detecting the toxicity of pesticides, including TNT and dinitrotoluenes (byproducts of TNT), has recently been reported^9, 27–30^. We utilized this cell line to detect the genotoxicity of dicrotophos and to address the molecular mechanism of 3,3′-dichlorobenzidine-mediated toxicity^30, 31^. Recently, we proved the role of miRNA in inhibiting DNA repair proteins in HepG2 cells- treated with 4-aminobiphenyl, a well-known carcinogen that induces bladder cancer^32^. In this study, we have shown that TNT has a significant inhibitory effect on the growth of HepG2 and Hep3B cells. HepG2 and Hep3B cells are of great relevance for detecting cytotoxic and genotoxic substances^23^; therefore, these cell lines were utilized to elucidate the molecular mechanism of TNT-induced apoptosis through an ROS-dependent destructive cycle involving DNA damage, ER stress and mitochondrial dysfunction.

RNA-seq provides a far more precise measurement of levels of transcripts than other traditional methods^33^. With this method, we successfully measured the change of transcriptome responses in zebrafish embryos before and after triadimefon^34^. Accordingly, in this study, RNA-seq was utilized to assess the transcriptome responses in cells under TNT stress. KEGG analysis revealed that oxidative phosphorylation was one of the enriched transcriptional responses. Many genes related to oxidative phosphorylation were dysregulated after the TNT treatment (Supplementary Tables S4 and S5), suggesting that mitochondrial function could be affected by TNT. Mitochondrial dysfunction in the form of oxidative stress and mutations can contribute to the pathogenesis of various neurodegenerative diseases such as Parkinson’s disease (PD), Alzheimer’s disease (AD), and Huntingon’s disease (HD)^35^. When the cells were treated with 80 μM TNT, the most significant response was the dysregulation of genes related to AD and HD, which could have resulted from mitochondrial dysfunction, as suggested by Arun *et al*.^35^, who reported on the role of mitochondrial dysfunction in the pathogenesis of these diseases.

Several recent studies have shown that the generation of ROS by diverse cell-death stimuli not only turns on the cell death signals but also leads directly to DNA damage^36^. In this study, the comet assay and γ-H2AX protein expressions results clearly showed that TNT could cause DNA damage in HepG2 cells, and DNA damage was alleviated when the cells were pretreated with NAC. p53 is a major cellular stress sensor that is activated in response to DNA damage such as telomere dysfunction and other adverse stimuli such as ROS^37^. Chung *et al*. reported that DNA damage induced by drugs such as sinularin can initiate p53-dependent and p53-independent pathways^38^. In this study, a significant increase in p53 protein levels was observed in the HepG2 cells. We also observed DNA damage, ER stress, mitochondrial dysfunction, and apoptosis in Hep3B cells, a p53-null cell line, when the cells were treated with TNT. These results suggest that TNT-induced cell apoptosis occurs independently of p53 in Hep3B cells. Given that DNA damage usually comes with oxidative stress and that ROS could be involved in leading to mitochondrial dysfunction^38^, we used JC-1 dye to stain viable mitochondria and flow cytometry to measure mitochondrial membrane potential. We indeed showed that TNT can disrupt mitochondrial membrane potential. Two sites in the mitochondria, complex I and complex III, have been suggested as the major sites for ROS production^39^. Zhou *et al*. showed that ROS production increased in human leukemia cell lines when complex III activity was decreased by miltirone^12^. Similarly, we detected the dysregulation of genes related to complex I and complex III in cells after TNT treatment, although the activity of these complexes was not measured. Therefore, we reasonably suggested that ROS mainly originated from mitochondria when the levels of mitochondrial dysfunction were rescued in cells pretreated with NAC.

In this study, we showed that TNT could induce a pronounced increase of ROS and lead to apoptosis. A critical question arises regarding the apoptotic mechanism by which ROS elevation occurs during TNT treatment. Woo *et al*. has been demonstrated that apoptosis is related to MMP depolarization and that the permeabilization of outer mitochondrial membranes is a critical step in apoptosis^40^. Our results demonstrated that TNT induced MMP depolarization. Furthermore, the loss of MMP was attenuated when cells were pretreated with NAC, indicating that ROS participates in mitochondrial dysfunction. Bcl-2, which belongs to the anti-apoptotic family, can bind to Bax and prevent the permeabilization of the outer mitochondrial membrane^41^. TNT decreased the expression of Bcl-2 and increased the expression of Bax, leading to an increase in the Bax/Bcl-2 ratio. In addition, TNT significantly decreased the expression of pro-caspase-9 and -3 and increase cleaved caspase-9 and -3 respectively, and increased the activity of caspase-3/7. These results indicate that TNT could induce the mitochondria-related apoptotic pathway. However, the above results were reversed when these cells were pretreated with NAC, indicating that ROS could play an important role in apoptosis.

The activation of unfolded protein response (UPR) plays a protective role in cells under ER stress. Physiological processes that demand a high rate of protein synthesis and secretion must sustain activation of the UPR’s adaptive programs without triggering cell death pathways. However, the activation of UPR by excessive ER stress can convert its role to cytotoxic by activation of multiple apoptotic pathways in mammalian cells cells^42^. IRE1-α, eIF2α and PERK constitute the core stress regulators of the UPR and transduce signals from the ER to the cytoplasm and nucleus after ER stress^42^. PDI is an ER chaperon, that is responsible for the formation of disulfide bonds in proteins^43^. BiP, which is an intracellular chaperon, regulates the unfolded protein response during ER stress^44^. Ero1-Lα is an upstream signal of ER stress and has been conjectured to hyperoxidize the ER lumen and cause cytotoxic ROS production, leading to cell death^45^. Another mechanism was associated with the role of Bax in CHOP-induced apoptosis. Additionally, CHOP, a transcription factor, can decrease Bcl-2 transcription and promote ROS production^46^, as our results in the present study have shown. These results implicated the involvement of ER stress in TNT-induced apoptosis.

Much evidence has shown that ROS can disturb ER protein folding and induce ER stress. In our results, we found that levels of ER stress can be attenuated by NAC. Hence, ER stress may be associated with TNT-induced oxidative stress. To investigate the possible role of ER stress in TNT-induced apoptosis, the knockdown of CHOP was used to alleviate the ER stress. These treated cells significantly attenuated the TNT-mediated apoptosis.

**Figure 7:**
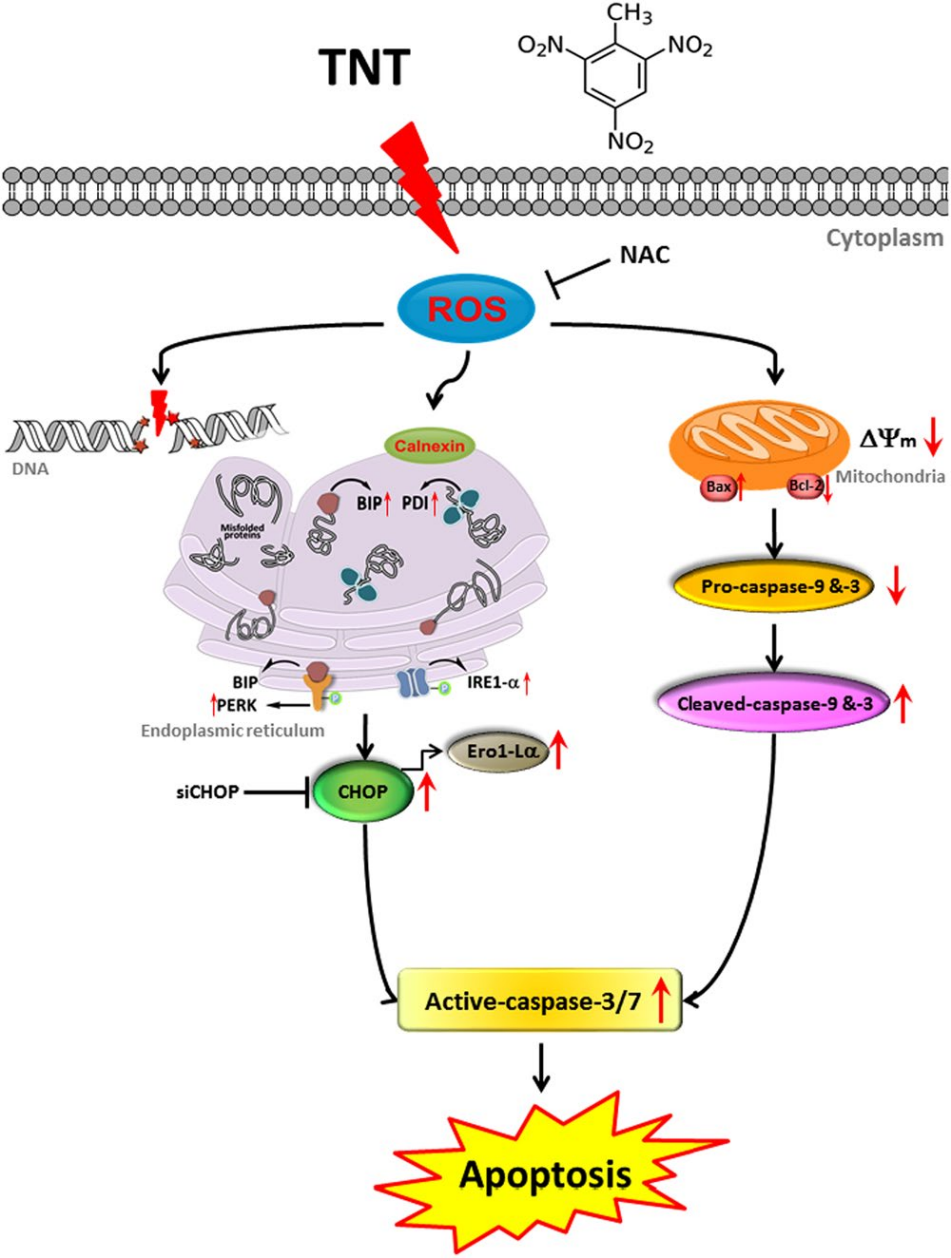
The schematic representation of proposed mechanisms according to TNT-induced apoptosis in HepG2 cells.

In summary, the present findings demonstrate that TNT effectively induced apoptosis through ROS-dependent DNA damage, ER stress and mitochondrial dysfunction. We provided the detailed mechanistic basis for TNT-induced cell death under oxidative stress. Besides, NAC could significantly increase the viability of TNT-treated cells (Fig. 5). Finally, this study presented the mechanisms of TNT-induced HepG2 cell apoptosis through ROS-dependent mitochondria and ER stress pathways (Fig. 7).

## Materials and Methods

### Materials

The TNT (99.9% purity) used in the present study was provided by the Taiwan Explosive Chemical Laboratory, Combined Logistics Forces, Taiwan. The N-acetylcysteine (NAC) and dimethyl sulfoxide (DMSO) came from Sigma-Aldrich (St. Louis, MO, USA).

### Cell culture

HepG2 and Hep3B cells were cultured in Dulbecco’s modified Eagle medium (DMEM, Lonza, BE12-614, Barcelona, Spain) containing 10% (v/v) fetal bovine serum (Lonza), 2 mM l-glutamine, 50 U/mL penicillin, and 0.1 mg/mL streptomycin (Sigma) in a humidified incubator with 5% CO_2_ at 37 °C.

### Cell treatment

The cells were cultured in 10-cm^2^ dishes until 70% confluence was reached. The medium was then replaced with a fresh serum-free medium containing the indicated TNT stock solution. Different concentrations of TNT (0–100 μg/mL) were dissolved in 0.5% DMSO. The negative controls were exposed to 0.5% DMSO. The treated cells were exposed to the TNT stock solution for a total of 24 h or 48 h.

### Cell viability

The cell viability of HepG2 and Hep3B cells was detected using Cell Counting Kit-8 (CCK-8 Donjindo Molecular Technologies, Inc., MD, USA) according to the manufacturer’s instruction. Cells in a 96-well plate (5 × 10^4^ cells/well) were incubated with or without various concentrations of TNT for 12 h or 24 h. Thereafter, 10 μL of CCK-8 solution was added to each well, and the cells were incubated at 37 °C for 1–4 h. The absorbance was measured at 450 nm using a microplate spectrofluorometer (Mark; Bio-Rad, Hercules, CA, USA).

### Comet assay

The comet assay was performed under alkaline conditions using our previously described methods^47^. At least 300 images were randomly selected from each sample and analyzed for DNA damage with the Comet IV computer software (Perceptive Instruments, UK). The tail moment comet parameter (mean ± SD) was used as an indicator of DNA damage.

### Total RNA isolation

The total RNA from the HepG2 cells was obtained using TRIzol Reagent (Invitrogen, Carlsbad, CA, USA) following the manufacturer’s instructions. Briefly, after the cells had been treated cells for 24 h, they were washed with 1 mL TRIzol Reagent and 200 μL of chloroform during a short incubation. After mixing vigorously, the solution was centrifuged at 13,000 × g for 20 min. The RNA was then precipitated in isopropyl alcohol, washed with 75% ethanol, pelleted, and resolubilized in nuclease-free water. RNA quantity and purity were measured spectrophotometrically (BioPhotometer, Eppendorf). The samples were considered suitable for further processing if the A260/A280 ratios were between 1.8 and 2.0. RNA integrity was determined with 1.8% agarose electrophoresis gel.

### Gene expression profiles: The RNA sequencing

The RNA sequencing (RNA-seq) was performed according to our previously described methods^34^. The total extracted RNA was first treated with DNase I to degrade any possible DNA contamination. Poly-A mRNA was isolated using oligo (dT) magnetic beads and then fragmented. The cleaved RNA fragments were transcribed into first-strand cDNA using reverse transcriptase and random hexamer primers, which was followed by second-strand cDNA synthesis. The double -stranded cDNA was further subjected to end-repair, phosphorylation, 3′-adenylation, and adaptor ligation in sequence. Adaptor ligated fragments were selected according to the size (250–350 bp), and cDNA fragments in the desired range were extracted from the gel. The fragments were enriched by PCR amplification. After qualitative and quantitative analysis by an Agilent 2100 Bioanalyzer and the ABI StepOnePlus Real-Time PCR System, respectively, the cDNA libraries were subjected to RNA-seq via Illumina HiSeq. 2000 (BGI, Shenzhen, China).

### Analysis of differential gene expression

The original image data generated by the sequencer were converted into sequences. After the filtration of the low-quality reads, the raw reads were cleaned up by removing adapter sequences based on the illumine pipeline. Gene expression profiling was measured by mapping clean reads onto assembled sequences using SOAP software. Gene expression levels were calculated by using the reads per kilobase of transcript sequence per million mapped reads (RPKM)^48^. This study followed a modified version of Audic’s method screen for the differentially expressed genes (DEGs)^49^. The false discovery rate (FDR) method was used to determine the threshold of the *p*-value. We used an FDR of ≦0.001 and an absolute value of log2 ratio ≧1 as the threshold to judge the significance of gene expression difference.

### Pathway analysis

All genes must cooperate with each other to exercise their biological functions. We mapped all DEGs onto terms from the Kyoto Encyclopedia of Genes and Genomes (KEGG) database (http://www.genome.jp/kegg/pathway.html), where a *q*-value of <0.05 indicated significantly enriched terms in DEGs.

### Caspase-3/7 activity assay

Cells from each treatment group were harvested after 24 h and caspase activity was detected by a Caspase-Glo 3/7 assay kit (Promega, Taipei, Taiwan). Briefly, 100 μL of Caspase-Glo 3/7 reagent was added to each well of the plates, followed by mixing for 1 min. Subsequently, these plates were incubated at room temperature for 2 h. Absorbance values were measured with a microplate reader at 405 nm (Synergy HTX Multi-Mode Reader; Biotek, Taipei, Taiwan).

### Detection of intracellular ROS

The production of intracellular ROS was detected using fluorescent dye 2′,7′- dichlorodihydrofluorescein diacetate (H_2_DCFDA, Sigma, USA). Briefly, 5 × 10^5^ cells/well cultured in a 6-well plate were incubated with or without different concentrations (HepG2: 0, 30, or 80 μM; Hep3B:0, 50, 60 μM) of TNT for the indicated time (with 100 μM H_2_O_2_ as a positive control). The cells were stained with 10 μM H_2_DCFDA for 30 min. Then the cells were examined under a fluorescence microscope, and the fluorescence intensity generated in the cells was detected using a fluorescence plate reader at an excitation wavelength of 488 nm and an emission wavelength of 525 nm.

### Western blot analysis

Western blot analysis was performed as previously described^32^. The culture medium was replaced with a new medium when the HepG2 cells were 70% confluent, then, the cells were exposed to various concentrations of TNT for 24 h. Subsequently, the cells were washed twice with PBS and then lysed. From each sample, 50 μg protein was subjected to 10% SDS polyacrylamide gel electrophoresis, transferred to PVDF membranes, and blocked with 5% skim milk at room temperature for 1 h. After blocking, the membrane was incubated with antibodies against pro-caspase-9, pro-caspase-3, cleaved-caspase-9, cleaved-caspase-3, β-actin, calnexin, PDI, BIP, IRE1-α, PERK, Ero1-Lα, CHOP, Bax, Bcl-2, phosphorylation of IRE1, phosphorylation of PERK and phosphorylation of eIF2α (Cell Signaling, USA) for 1 h. Then, the membranes were washed with 0.1% PBST (PBS and 0.05% Tween 20) and incubated with secondary antibodies conjugated to horseradish peroxidase for 1 h. Bands were detected after chemiluminescent HRP (Immunobilon Western, Millipore) was added. The band density was measured with ImageQuant-TL7.0 software (GE Healthcare).

### Mitochondrial Membrane Potential (MMP) measurement

The disruption of MMP was measured using fluorochrome dye JC-1 by flow cytometry as reported by Zhou *et al*.^12^. After the cells were treated with different concentration of TNT, the cells were harvested and washed twice with PBS. Then, the cells were treated with JC-1 for 30 min and analyzed in a flow cytometer. Each group acquired more than 10,000 individual cells.

### Validation by reverse transcription quantitative PCR

To confirm the expression results of the mRNAs obtained from RNA-seq, SYBR Green I® Quantitative Real-Time PCR (qRT-PCR) was performed with an IQ-5 Real-Time PCR System (BIO RAD). All of the primer sequences used for qRT-PCR are listed in Supporting Information Table S6. In brief, a total of 10 ng RNA for each sample was used to perform qRT-PCR. cDNA was synthesized from the total RNA using a qScript cDNA Synthesis Kit (Quanta BioSciences, Gaithersburg, MD, USA) according to the manufacturer’s instructions. Subsequently, amplifications were carried out under the following conditions: 95 °C for 10 min (initial denaturation), 35 combined cycles at 95 °C for 30 s (denaturation), 57 °C for 45 s (annealing), and 72 °C for 1 min (extension). Fluorescence was measured in real time. The cycle threshold (Ct) values were calculated using the LightCycler3 data analysis software. The fold expression or repression of the target gene relative to the internal control gene in each sample was calculated by following the ΔΔCt model. All the samples were analyzed in triplicate, and the mean value of these triplicate measurements was used to calculate the mRNA transcriptions.

### The siRNA transfection

The cells were seeded in 6-well plates at a density of 5 × 10^5^ cells/mL and allowed to reach approximately 70% confluence on the day of transfection. The small interfering RNA (siRNA) against CHOP was purchased commercially from Sigma, USA. The cells were transfected with CHOP siRNA (100 nM) using Lipofectamine 2000 (Invitrogen, Carlsbad, CA, USA) according to the manufacturer’s instructions. After 24 h, the cells were treated with TNT for 24 h and examined by Western blot.

## Electronic supplementary material

Supplemental Figure 1–2 and Table 3–6

Supplemental Table 1

Supplemental Table 2
