# Supplementary material for: 2,4,6-Trinitrotoluene Induces Apoptosis via ROS-Regulated Mitochondrial Dysfunction and Endoplasmic Reticulum Stress in HepG2 and Hep3B Cells
Source: Sci Rep. 2017 Aug 15;7:8148. doi: 10.1038/s41598-017-08308-z (PMC5557873; doi:10.1038/s41598-017-08308-z)
Supplement: Supplementary file 1 — Supplemental Figure 1–2 and Table 3–6 [file 41598_2017_8308_MOESM1_ESM.pdf]

2,4,6-Trinitrotoluene Induces Apoptosis via ROS-Regulated Mitochondrial Dysfunction and Endoplasmic Reticulum Stress in HepG2 and Hep3B Cells

Hung-Yu Liao, Chih-Ming Kao, Chao-Ling Yao, Po-Wei Chiu, Chun-Chen Yao, and Ssu-Ching Chen

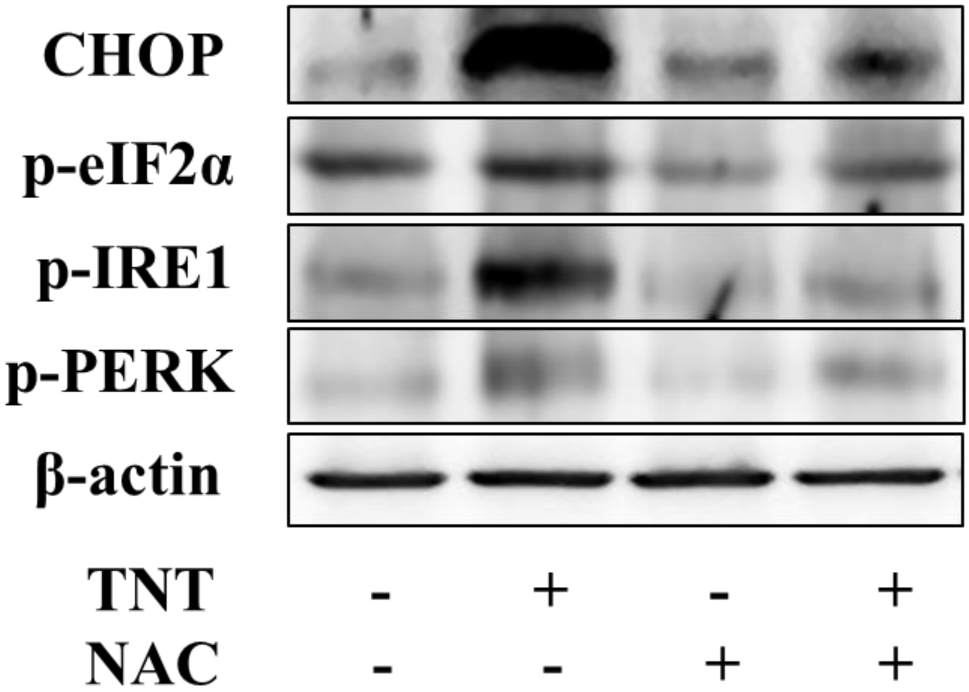

Supplemental Figure 1. ROS-dependent ER stress was involved in the TNT-mediated ROS production. (A) Hep3B cells were treated with 60 μM TNT for 24 h in the presence or absence of 10 mM NAC, given the expression of ER stress-related proteins. The bands were excised from different gels that were run under the same electrophoresis condition.

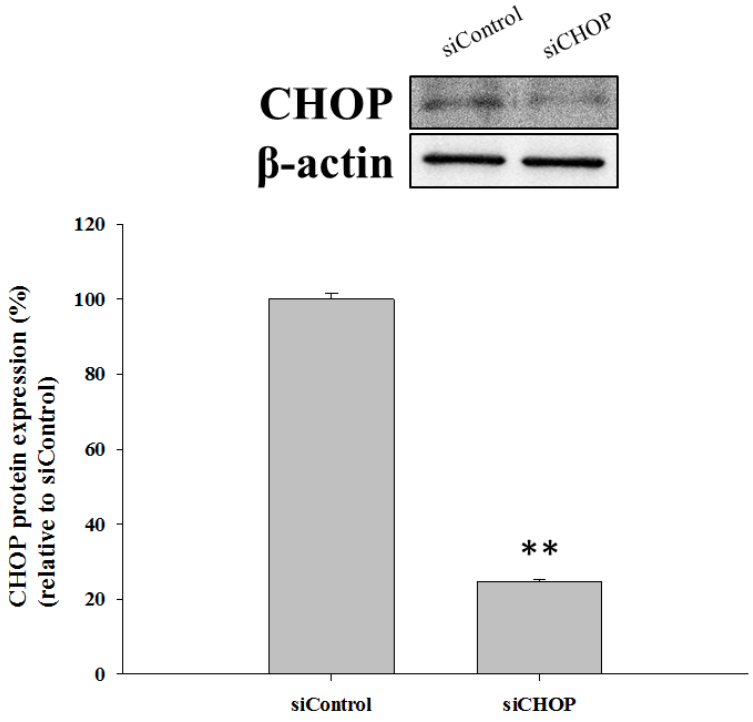

Supplemental Figure 2. HepG2 cells were transfected with CHOP siRNA for 24h, and analysed by western blot; n = 3. The data were presented as mean ± SD. The differences were significant at *P < 0.05, **P < 0.01.

Supplemental Table 3. Comparison of RNA-seq results by quantitative PCR on differentially expressed genes

Gene	Fold change (log2)	
	RNA-seq	q-PCR
GADD34 *(PPP1R15A)*	4.14	12±0.12
ATF3	1.72	6±0.6
BIP *(HspA5)*	5.6	1.4±0.2
HERP *(HERPUD1)*	0.5	2.1±0.8
EDEM1	3.2	2.3±0.3
CHOP *(DDIT3)*	2.2	4.6±0.8
ASNS	1	1.7±0.2
IGFBP	4.4	2.2±0.2
CYP1A1	5.8	4±0.6
ATP6V0A1	1.9	1.2±0.4
NDUFA1	-6	-4.2±0.6
SDHD	-1	-1.3±0.3
COX6C	-2.6	-2.4±0.4
GRP94 *(HSP90B1)*	3	2±0.3

The values represent the mean ± SD and were derived from at least three independent experiments. Triplicate measurements were performed for each experiment. All the data

$P < 0.05$.

Supplemental Table 4.

#	Pathway	DEGs with pathway annotation (1309)	All genes with pathway annotation (17252)	Pvalue	Qvalue
1	Oxidative phosphorylation	30 (2.29%)	148 (0.86%)	5.88379e-07	0.0001441529
2	Alzheimer's disease	39 (2.98%)	234 (1.36%)	2.565723e-06	0.0003143011
3	Ribosome	27 (2.06%)	147 (0.85%)	1.460253e-05	0.0011925399
4	Parkinson's disease	29 (2.22%)	167 (0.97%)	2.174453e-05	0.0013318525
5	Huntington's disease	39 (2.98%)	265 (1.54%)	4.964504e-05	0.0024326070
6	Metabolism of xenobiotics by cytochrome P450	17 (1.3%)	101 (0.59%)	0.001484609	0.0606215342
7	Complement and coagulation cascades	25 (1.91%)	181 (1.05%)	0.002573143	0.0839549095
8	Phosphatidylinositol signaling system	22 (1.68%)	155 (0.9%)	0.003223227	0.0839549095
9	Glycerolipid metabolism	15 (1.15%)	91 (0.53%)	0.003380644	0.0839549095
10	Metabolic pathways	252 (19.25%)	2847 (16.5%)	0.003426731	0.0839549095
11	Dorso-ventral axis formation	12 (0.92%)	67 (0.39%)	0.004193373	0.0933978532
12	Ubiquitin mediated proteolysis	28 (2.14%)	230 (1.33%)	0.008825692	0.1801912117
13	Pyrimidine metabolism	19 (1.45%)	144 (0.83%)	0.01268108	0.2389895846
14	Steroid hormone biosynthesis	11 (0.84%)	69 (0.4%)	0.01427793	0.2498637750
15	Maturity onset diabetes of the young	6 (0.46%)	29 (0.17%)	0.01975013	0.2879070950

16	Lysine degradation	14 (1.07%)	102 (0.59%)	0.02176809	0.2879070950
17	Adherens junction	21 (1.6%)	173 (1%)	0.02186023	0.2879070950
18	Regulation of actin cytoskeleton	58 (4.43%)	586 (3.4%)	0.02225297	0.2879070950
19	Citrate cycle (TCA cycle)	7 (0.53%)	38 (0.22%)	0.02251832	0.2879070950
20	Glycerophospholipid metabolism	17 (1.3%)	133 (0.77%)	0.02350262	0.2879070950
21	Selenocompound metabolism	5 (0.38%)	23 (0.13%)	0.02658661	0.2932811292
22	Ascorbate and aldarate metabolism	6 (0.46%)	31 (0.18%)	0.02691747	0.2932811292
23	Fat digestion and absorption	11 (0.84%)	76 (0.44%)	0.02775248	0.2932811292
24	Lipoic acid metabolism	4 (0.31%)	16 (0.09%)	0.02872958	0.2932811292
25	Drug metabolism - cytochrome P450	12 (0.92%)	89 (0.52%)	0.03620829	0.3548412420
26	Inositol phosphate metabolism	13 (0.99%)	101 (0.59%)	0.04172799	0.3792422148
27	Glycolysis / Gluconeogenesis	11 (0.84%)	81 (0.47%)	0.04179404	0.3792422148
28	Glyoxylate and dicarboxylate metabolism	6 (0.46%)	35 (0.2%)	0.04595226	0.4020822750
29	Pentose and glucuronate interconversions	8 (0.61%)	54 (0.31%)	0.04961353	0.4191487879

Supplemental Table 5. Differential Pathway detected in TNT (80 μM)

#	Pathway	DEGs with pathway annotation (4865)	All genes with pathway annotation (17252)	Pvalue	Qvalue
1	Alzheimer's disease	107 (2.2%)	234 (1.36%)	7.073067e-09	1.803632e-06
2	Huntington's disease	112 (2.3%)	265 (1.54%)	5.406575e-07	6.893383e-05

3	Ribosome biogenesis in eukaryotes	59 (1.21%)	124 (0.72%)	3.387542e-06	2.879411e-04
4	Oxidative phosphorylation	67 (1.38%)	148 (0.86%)	6.618594e-06	4.219354e-04
5	Insulin signaling pathway	96 (1.97%)	235 (1.36%)	1.835939e-05	9.363289e-04
6	Ribosome	64 (1.32%)	147 (0.85%)	4.695647e-05	1.995650e-03
7	Parkinson's disease	70 (1.44%)	167 (0.97%)	9.233666e-05	3.363693e-03
8	Ubiquitin mediated proteolysis	91 (1.87%)	230 (1.33%)	0.0001208199	3.851134e-03
9	Phosphatidylinositol signaling system	65 (1.34%)	155 (0.9%)	0.0001587002	4.496506e-03
10	Metabolic pathways	880 (18.09%)	2847 (16.5%)	0.0002596131	6.620134e-03
11	Inositol phosphate metabolism	45 (0.92%)	101 (0.59%)	0.0003176133	7.362854e-03
12	Protein processing in endoplasmic reticulum	92 (1.89%)	246 (1.43%)	0.001026296	2.130086e-02
13	Non-small cell lung cancer	38 (0.78%)	86 (0.5%)	0.001085926	2.130086e-02
14	Endocytosis	144 (2.96%)	413 (2.39%)	0.001645516	2.997190e-02
15	Cell cycle	70 (1.44%)	183 (1.06%)	0.001983976	3.372759e-02
16	Hepatitis C	68 (1.4%)	178 (1.03%)	0.002364879	3.404170e-02
17	Tight junction	150 (3.08%)	436 (2.53%)	0.002439795	3.404170e-02
18	Prostate cancer	59 (1.21%)	151 (0.88%)	0.002445115	3.404170e-02
19	Fructose and mannose metabolism	29 (0.6%)	64 (0.37%)	0.002556393	3.404170e-02
20	Regulation of actin cytoskeleton	196 (4.03%)	586 (3.4%)	0.002669937	3.404170e-02

21	Lipoic acid metabolism	10 (0.21%)	16 (0.09%)	0.00435793	5.291772e-02
22	Endometrial cancer	35 (0.72%)	84 (0.49%)	0.005415399	5.685964e-02
23	Colorectal cancer	35 (0.72%)	84 (0.49%)	0.005415399	5.685964e-02
24	Proteasome	23 (0.47%)	50 (0.29%)	0.00545178	5.685964e-02
25	Pancreatic cancer	41 (0.84%)	102 (0.59%)	0.0058959	5.685964e-02
26	Chronic myeloid leukemia	51 (1.05%)	132 (0.77%)	0.005970184	5.685964e-02
27	Glioma	46 (0.95%)	117 (0.68%)	0.006020432	5.685964e-02
28	Glycerophospholipid metabolism	51 (1.05%)	133 (0.77%)	0.007076486	6.360416e-02
29	Adherens junction	64 (1.32%)	173 (1%)	0.007233414	6.360416e-02
30	Pyrimidine metabolism	54 (1.11%)	144 (0.83%)	0.00951059	7.988206e-02
31	Epstein-Barr virus infection	105 (2.16%)	305 (1.77%)	0.009711152	7.988206e-02
32	Dorso-ventral axis formation	28 (0.58%)	67 (0.39%)	0.01150448	9.167632e-02
33	Glycerolipid metabolism	36 (0.74%)	91 (0.53%)	0.01253363	9.685078e-02
34	Renal cell carcinoma	41 (0.84%)	107 (0.62%)	0.01480232	1.110174e-01
35	ErbB signaling pathway	53 (1.09%)	145 (0.84%)	0.01737806	1.266116e-01
36	Butanoate metabolism	22 (0.45%)	53 (0.31%)	0.02545113	1.802788e-01
37	Fatty acid biosynthesis	7 (0.14%)	12 (0.07%)	0.02768269	1.907861e-01
38	Vasopressin-regulated water reabsorption	29 (0.6%)	75 (0.43%)	0.03204072	2.115289e-01

39	Basal transcription factors	26 (0.53%)	66 (0.38%)	0.03235148	2.115289e-01
40	Biosynthesis of unsaturated fatty acids	15 (0.31%)	34 (0.2%)	0.0341744	2.160657e-01
41	Long-term potentiation	44 (0.9%)	122 (0.71%)	0.03533915	2.160657e-01
42	mTOR signaling pathway	35 (0.72%)	94 (0.54%)	0.03558729	2.160657e-01
43	VEGF signaling pathway	49 (1.01%)	138 (0.8%)	0.0364476	2.161427e-01
44	RNA polymerase	19 (0.39%)	46 (0.27%)	0.03821451	2.214705e-01

Supplemental Table 6. Primer list

Name	Sequence
GADD34	+467 : TCCTCTGGCAATCCCCCATA
	-570 : TGGTTTTCAGCCCCAGTGTT
ATF3	+1347 : GCTGTCACCACGTGCAGTATCTCA
	-1416 : TCTGTCCTCCTCTTGCTGACAAGC
BIP	+1696 : CTGGGTACATTTGATCTGACTGG
(HspA6)	-1931 : TCCTTGAGCTTTTTGTCTTCCT
HERP	+787 : CTCCAGCCCCTATTCACAAC
(HERPUD1)	-957 : TCCAATCCAACCAATCTCG
EDEM	+1678 : GAAATGAAAGGGGACAGAAG
	-1945 : GCCAGCAAAGTGAAGAGAC
CHOP	+373 : CAGAACCAGCAGAGGTCACA
(DDIT3)	-571 : AGCTGTGCCACTTTCCTTTC
ASNS	+490 : ATCACTGTCGGGATGTACCC
	-692 : CTTCAACAGAGTGGCAGCAA
IGFBP	+765 : TATGATGGCTCGAAGGCTCT
	-988 : TAGACGCACCAGCAGAGTC
CYP1A1	+634 : CTCCATTGCCTCTGACCCAG
	-943 : CAGGGAAGGGTTGGGTAGGT
ATP6V0A1	+1330: GAGAGATAAATCCAGCTCCGT
	-1549 : GTGTACATGGAGAACACACC
NDUFA1	+141 : GATGTGGTTCGAGATTCTCC
	-317 : CGATCAACTCCAGAGATGCG

SDHD	+79 : AACGAGATGGCGGTTCTCTG
	-267 : CTTGTCCAAGGCCCAATGGT
COX6C	+401 : AACTACCATGGCTCCCGAAG
	-618 : AGATACCAGCCTTCCTCATC
GRP94	+766 : TTGGTGTCGGTTTCTATTCC
(HSP90B1)	-843 : GCTGGGTATCGTTGTTGTG
